# Let's get moving! Improving physical activity amongst rehabilitation patients; a quality improvement project

**DOI:** 10.1192/bjo.2021.576

**Published:** 2021-06-18

**Authors:** Ruth Rowland, Laura Somerville, Sarah Dorman, Mark Finnerty

**Affiliations:** Downshire Hospital

## Abstract

**Aims:**

This Quality Improvement Project aimed to improve physical activity amongst patients in a 16-bedded, low secure unit in the Downshire Hospital, Northern Ireland. We introduced an exercise programme with the aim of increasing minutes of physical activity per week. Secondary outcome measures were weight, mood and energy levels.

This project took place in the context of COVID-19 restrictions having reduced opportunities for off-ward activity and staff noting subsequent deconditioning and weight gain amongst the patient cohort. Cohort consisted largely of patients with a severe mental illness, many of whom had physical health co-mobridities.

**Method:**

This project included all patients in the 16-bedded unit.

Baseline data were collected prior to programme introduction, including weekly activity levels and weights. A questionnaire explored patient confidence and attitude towards physical activity.

Focus groups were held with patients and staff in order to identify how best to introduce the programme, discuss content, and identify potential barriers.

We introduced an eight-week programme of weekly, thirty-minute, mixed ability exercise sessions. These were led collectively by the multi-disciplinary team. Patients actively participated in programme design; choosing session soundtracks and contributing to content planning.

Likert scales were used to measure self-report mood and energy levels pre- and post-session. Staff engaged in a weekly post session de-brief, where challenges were identified and solutions suggested. Weekly qualitative feedback was sought from participants. The sessions were thus developed and adapted according to patient and staff feedback over the programme's course.

Following the 8-week programme, activity levels and weight were re-measured and compared to baseline. Pre-programme questionnaires were also repeated.

**Result:**

Patients reported increased enjoyment and confidence engaging in physical activity, as well as improved overall self- confidence and a sense of pride and ownership of the sessions.

Staff reported a more cohesive team environment, greater sense of work-place fulfilment and improved therapeutic relationships.

Comparing pre and post session ten-point-Likert scales showed a 153% mean increase in self-rated energy levels and a 98% mean increase in self-rated mood. This reflected a mean score increase of 3.8 in both.

Minutes of physical activity per week increased for all session participants, although remained below national guidance.

Weight reduction did not occur.

**Conclusion:**

Exercise benefits not only physical health, but also emotional and psychological well-being. This project demonstrates how introduction of a weekly ward-based exercise class can offer this as well as improving working environment, team cohesion and therapeutic relationships. Weight reduction may be observed in the longer term.

